# NK Cell Precursors in Human Bone Marrow in Health and Inflammation

**DOI:** 10.3389/fimmu.2019.02045

**Published:** 2019-08-28

**Authors:** Federica Bozzano, Carola Perrone, Lorenzo Moretta, Andrea De Maria

**Affiliations:** ^1^Ospedale Pediatrico Bambin Gesù, Rome, Italy; ^2^Centro di Eccellenza per la Ricerca Biomedica, Università di Genova, Genoa, Italy; ^3^Clinica Malattie Infettive, Ospedale Policlinico S. Martino IRCCS, Genoa, Italy; ^4^Dipartimento di Scienze Dell Salute, Università Degli Studi di Genova, Genoa, Italy

**Keywords:** NK cells, CD34+ precursors, inflammation, common lymphoid precursor, DNAM-1, CXCR4

## Abstract

NK cells are generated from hematopoietic stem cells (HSC) residing in the bone marrow (BM), similar to other blood cells. Development toward mature NK cells occurs largely outside the BM through travel of CD34+ and other progenitor intermediates toward secondary lymphoid organs. The BM harbors multipotent CD34+ common lymphoid progenitors (CLPs) that generate T, B, NK, and Dendritic Cells and are devoid of erythroid, myeloid, and megakaryocytic potential. Over recent years, there has been a quest for single-lineage progenitors predominantly with the objective of manipulation and intervention in mind, which has led to the identification of unipotent NK cell progenitors devoid of other lymphoid lineage potential. Research efforts for the study of lymphopoiesis have almost exclusively concentrated on healthy donor tissues and on repopulation/transplant models. This has led to the widely accepted assumption that lymphopoiesis during disease states reflects the findings of these models. However, compelling evidences in animal models show that inflammation plays a fundamental role in the regulation of HSC maturation and release in the BM niches through several mechanisms including modulation of the CXCL12-CXCR4 expression. Indeed, recent findings during systemic inflammation in patients provide evidence that a so-far overlooked CLP exists in the BM (Lin^−^CD34^+^DNAM-1^bright^CXCR4^+^) and that it overwhelmingly exits the BM during systemic inflammation. These “inflammatory” precursors have a developmental trajectory toward surprisingly functional NK and T cells as reviewed here and mirror the steady state maintenance of the NK cell pool by CD34^+^DNAM-1^−^CXCR4^−^ precursors. Our understanding of NK cell precursor development may benefit from including a distinct “inflammatory” progenitor modeling of lymphoid precursors, allowing rapid deployment of specialized Lin^−^CD34^+^DNAM-1^bright^CXCR4^+^ -derived resources from the BM.

## Introduction

Natural Killer (NK) cells are innate lymphoid cells (ILC) with potent cytotoxic effector activity, due to their constitutive expression of perforin and granzyme and ready ability to produce high amounts of IFNγ and other proinflammatory cytokines. Their original definition of “born natural killers”(NK) was due to their “perforin-armed” resting condition. Their activity encompasses multiple defense activities including detection and disposal of virus-infected or transformed cells, early detection of pathogens via pathogen-associated molecular patterns (PAMPs) that are recognized by an array of innate receptors (e.g., TLRs), recruitment other cells involved in immune responses thanks to the early secretion of chemokines (IL-8, RANTES, MIP1a, MIP1b) and cytokines (GM-CSF, IL-6, IL-1) upon PAMP recognition. Subsequently, however, their involvement in the regulation of downstream responses was recognized with continuous coordination of and support to downstream adaptive immune responses through crosstalk with dendritic cells (1, 2) and with T cells (3–5). Over the last 10 years, it became clear that conventional NK cells are part of an extended family of ILC which includes three additional groups of innate cells having remarkable functional parallels with known helper T (Th) cell subsets (6, 7). Within this family, distinctive transcription factor expression and cytokine production characterizes conventional NK cells (Eomes, IFNγ) from group 1 ILC (T-bet, IFNγ), group 2 ILC (Gata3, IL-5/IL-13), and group 3 ILC (Rorc, IL17/IL-22). Several properties of conventional NK cells, and in particular transcription factor expression, clearly set them apart from other ILC subsets (8, 9), and has led to suggest a distinction between “helper” ILCs (ILC1s, ILC2s, and ILC3s) and “cytotoxic” ILCs (NK cells) that parallels the CD4+ Th cells vs. CD8+ CTL duality (10).

The predominant sites of the human body in which NK cells are found include secondary lymphoid organs, bone marrow, liver, lungs, and decidua while an overall minority of body NK cells (<2%) circulates in peripheral blood where they represent 5–15% of blood lymphocytes. Contrary to adaptive T or B cells, their functional specificity does not include somatic rearrangements. Their wide array of activating receptors is germline encoded and delivers potent triggering signals upon recognition of distress ligands expressed by stressed healthy as well as infected or transformed cells (11). NK cell function is tightly regulated by a balance between activating stimuli delivered through activating molecules and inhibitory signals primarily by HLA class I-specific inhibitory receptors (12). Inhibitory signaling recognizes self and, in most instances, overrides routine minor activating distress in order to avoid self-destruction unless NK cell activity is needed to control overt cell infection or transformation (13).

Similar to other blood cells, NK cells are generated from hematopoietic stem cells (HSC) residing in the bone marrow. Following the first experimental evidence of the possibility to rescue mice from lethal irradiation, the bone marrow (BM) has been identified as the main source of HSC in the body with a first estimate of 1 in 10e4 BM spleen-colony forming cells (14, 15). HSC with the characteristics of self-renewal and multipotency, that are able to generate more differentiated precursors along the pathways toward production of erythrocytes, leucocytes and platelets, were indeed thereafter identified in BM (16–18), with a frequency of 1 in 10e5 BM cells (16). According to a strictly hierarchical “stem tree” view where all cells derive from a common ancestor, progressive steps of differentiation of HSC lead to the generation of progressively more oligopotent precursors toward all blood cell lineages. The classical model of hematopoiesis postulates that the earliest fate decision toward NK cells downstream of HSCs is represented by the divergence of lymphoid and myeloid lineages. Erythroid and megakaryocyte lineages branch off before the lymphoid–myeloid split. This step is followed by myeloid–lymphoid divergence in which common lymphoid progenitors (CLPs), and common myeloid progenitors are generated (19). Alternate possibilities of a less stringent stem-root developmental model have been pursued. Thus, there is considerable heterogeneity in reconstituting HSCs, with proof of a less defined hierarchical transition reflecting different propensities for lineage-fate decisions by distinct myeloid-, lymphoid- and platelet-biased HSCs (20, 21). The low level of agreement on some aspects of decisional fate of progeny development in humans is primarily due to different experimental settings. So far, studies have been heterogeneous with regard to different aspects that that include the use of either adult or fetal/newborn materials that may be inadequate for a coherent comparison of results, the different study settings comparing analysis of precursors at steady state vs. repopulation studies with a push toward tissue and body repopulation after transplantation, and finally the exclusive use of healthy donors with a lack of data derived from disease states, in which the developmental push toward differentiation and self-maintenance may more strongly reflect the influence of inflammatory signals and/or of peripheral need caused by accelerated cell turnover.

The purpose of this review is to briefly summarize the findings on classical NK cell precursors in the bone marrow and to recapitulate recent findings on alternate new precursor populations. Only a brief mention to ILC development will be provided, since this is out of the purpose of the present work and may be obtained elsewhere (7, 22).

## NK Cell Development and Intermediates in the BM

After the first description of multipotent Lin^−^CD34+CD45RA+CD10+CD38+ progenitors in the BM generating *in vitro* T, B, NK, and Dendritic Cells (23), it became clear that the BM was the primary site of where NK cell precursors dwell and may generate NK cells (24). In fact, neither the thymus nor the spleen seemed to be essential for NK cell growth as shown by NK cell persistence and preserved function in their absence (25–27). The role of postnatal as compared to fetal liver in NK cell generation was unclear at the time and still requires further studies in future). Early views on NK cell development considered the BM as the main site for NK precursor growth from HSC and also the site where progressive NK cell development takes place (24).

Early work on BM precursors provided evidence that CD7 expression on CD34^+^CD45RA^+^ HPCs enriches for NK cell precursors (28). Also co-expression of CD10 on BM CD34^+^ HPCs identified a CLPs generating NK cells (23). These progenitors lacked erythroid, myeloid, and megakaryocytic potential but contained a broad B, T, and NK cell and DC differentiation potential, suggesting that this population might correspond to the human postnatal common lymphocyte precursor (CLP). It was also clear that CD34^+^CD7^−^ and CD34^+^10^−^ HPCs also could generate NK cells, albeit with lower efficiency and with more stringent contact requirement with stromal cells (21, 23, 28, 29). Subsequent studies revealed that CD10 expression on progenitors is associated with a strong bias toward B cell potential with minimal T or natural killer (NK) cell potential (28, 30, 31). Thus, the stepwise process of lymphoid differentiation from multipotent HSC to the earliest lymphoid-primed multipotent progenitor (LMPP) in BM was not characterized by the expression of CD10 (23), but rather of L-selectin (CD62L) expression on CD3-CD14-CD19-(henceforth Lin^−^) CD34^+^CD10^−^ progenitors (28). These progenitors were devoid of erythroid or myeloid clonogenic potential corresponding to LMPP and had the ability to seed SLT and thymus through the CD62L homing signal (21, 32, 33). In the same BM setting, CD7 expression alone did not define lymphoid commitment, as a Lin^−^CD34+CD38–CD7+ population that had been identified as a LMPP in umbilical cord blood (UCB) (34) was not detected, and low CD7 expression in CD34^+^Lin^−^CD38^+^CD10^−^ cells was insufficient to define lymphoid restriction as erythroid progenitors could also be detected (28). In UCB, circulating CD34+CD45+CD7+CD10– precursors could generate cells of the three lymphoid lineages, however, with a skewed potential toward the T/natural killer (T/NK) lineages. In contrast, CD34(^+^)CD45RA(^hi^)Lin(^−^)CD10(^+^) HPCs predominantly exhibited a B-cell differentiation potential. Also, a culture of purified CD34+ derived from UCB (without further subset sorting) with SCF, FLT3, IL-7, and IL15 generates *in vitro* CD3^−^CD16^+^CD56^+^CD244^+^CD33^−^ myelomonocytes and highly immature CD3^−^CD16^+^CD56^+^CD244^+^CD33^−^ NK cells that are substantially devoid of cytotoxic activity and of IFNγ production, without growth of T cells or other lieages (35–37).

More recently, Renaux et al. provided evidence that Lin^−^CD34^+^CD38^+^CD123^−^CD45RA^+^CD7^+^CD10^+^CD127^−^ cells purified from BM or UCB represent the unipotent NK cell precursor devoid of potential toward other lymphoid lineages (37, 38). These precursors are also detected in adult tonsils and fetal tissues and are different from Lin^−^CD34^+^ CD38^+^CD123^−^CD45RA^+^CD7^+^CD10^−^CD127^+^ cells, which can undergo different fates including myeloid lineages, and also different from Lin^−^CD34^+^CD38^+^CD123^−^CD45RA^+^CD7^+^CD10^+^CD127^+^ cells that generated only lymphoid lineages (T, B, ILC, NK cells) (38). Thus, this confirms previous reports on the origin of ILC from CD34+ precursors il SLT or UCB (39, 40). ILC development still bears some areas of uncertainty with need of additional focus (6, 7). Indeed, CD127 expression has been shown to represent a requirement for the fate decision toward ILC development from upstream precursors, which still bear NK, T, and B cell potency (38), and is expressed on ILC but not on NK cells. However, it is transiently not expressed on early innale lymphoid progenitors (EILIP) in the BM (7, 41, 42) and is also lacking on the single-fate NKP, which is supposed to be downstream ILC developmental potency (38).

Overall, therefore, it is clear that the BM harbors, in addition to totipotent HSCs, more committed lymphoid precursors with the ability to generate NK cells, including LMPP, CLP, and single-cell NKP. Also, there are evidences that the developmental fates of NK cells and of “helper” ILCs are intertwined in general up to CD127 retention on CLP. The occurrence of a local NK cell development in BM is not disputed, however, at present is not quantified and poorly defined.

The concept that the BM could not be the predominant site of NK cell development developed after the first reports on secondary lymphoid tissue NK cell composition. Indeed, lymphnodes were harboring predominantly large numbers of CD56^bright^ NK cells adjacent to T-cell-rich areas (43). This led to experiments showing that NK cells with a CD56^dim^ phenotype developed from CD56^bright^ NK cells to take place in SLTs (44). Proof of this concept followed these observations and was substantiated by Freud et al. (45) with the description and characterization in lymphnodes of CD34+ CLP generating CD56^bright^ NK cells *in vitro* (45). Subsequent work confirmed the presence of CD34+ CLP in SLT and thymus, generating CD56^bright^ NK cells. Finally, MMLP and CLP have been recovered from PB and from UCB, and in general are believed to transit from BM through PB toward peripheral tissues for further development. These observations, therefore, supported the concept that the BM is the site where HSC are contained and is the origin of MLP and CSP, and that the vast majority of NK cells may be generated in peripheral tissues (e.g., SLT) as progenies from CLP traveling from the BM.

## BM Organization of the HSC Microenvironment

All the so-far described BM NK cell precursor populations have been investigated according to the assumption of an uncharacterized anatomical organization, in which HSC reside in the BM within specialized microenvironments or niches. Animal studies have contributed a wealth of information on hematopoietic organization of the marrow microenvironment. Quiescent HSCs reside in perivascular niches, in which different cell types express or release factors that promote HSC maintenance (46). Quiescent HSCs in mice BM associate specifically with small arterioles that are preferentially found in endosteal BM (47). The production of CXCL12 by cells present in the perivascular region, including stromal cells, sinusoidal endothelial cells, and mesenchymal progenitors, has been shown to support HSC retention. Accordingly, CXCL12 deletion in mice results in constitutive HSC mobilization (48).

**Figure 1:**
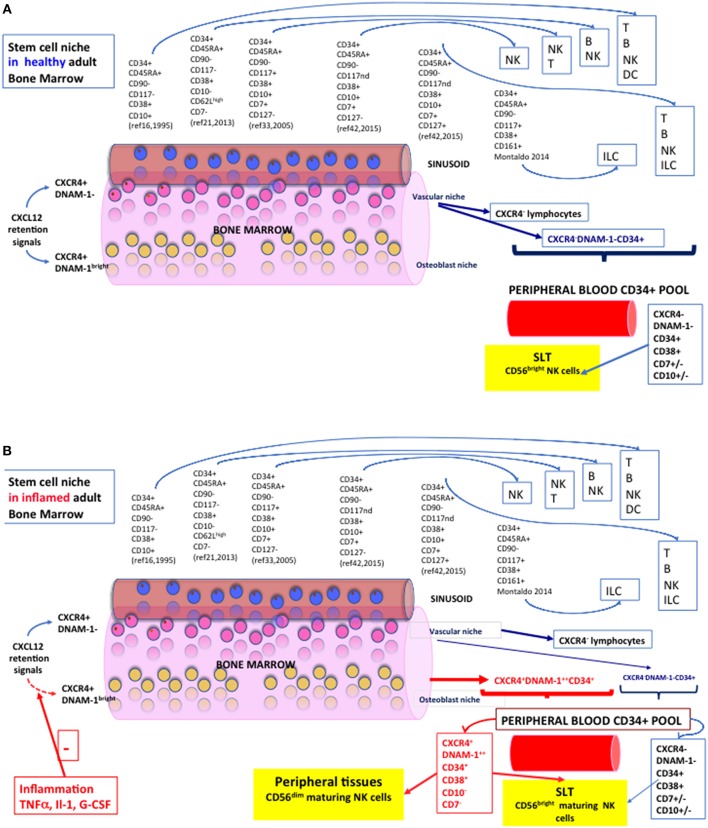
NK cell hematopoiesis in health and during systemic inflammation. A section of Bone Marrow with a sinusoid is represented. Vascular Niche in pink, osteoblast niche representation in yellow. Blue cells represent CXCR4- lymphoid or erythroid cells passively released in the sinusoid. A spectrum of the phenotypes of so far characterized CD34+ NK cell precursors is represented with reported progenies and is indicated by citation numbers. **(A)** Diagram of the Stem Cell Niche and NK cell precursors in Healthy Adult Bone Marrow. Lymphoid cells and precursors exiting passively from sinusoids are indicated by dark blue arrows and constitute the pool of CD34+ cells circulating in peripheral blood. A yellow box defines their trajectory toward SLT (CD62L+CCR7+) and the prevailing phenotype of NK cells grown under standard conditions *in vitro*. **(B)** The Stem Cell Niche and CD34+ NK cell precursors in Adult Bone Marrow during chronic inflammation. During chronic inflammation, inflammatory cytokines and mediators determine a reduction/shutdown of CXCL12 signaling within the BM niches, with decreased retention ability of CXCR4+ HSC and CLP that otherwise populate the BM but do not circulate in PB. Red arrows show “inflammatory” or “emergency” CD34+ cells exting the BM. A different composition of CD34+ peripheral blood pool is accordingly shown during inflammatory conditions (HIV, HCV, COPD, Tuberculosis, PAPA). Travel trajectories of Lin^−^CD34^+^DNAM-1^bright^CXCR4^+^ cells toward peripheral tissues are shown as these CD34+ CLPa express CX3CR1+, CXCR1+, CXCR3+, in addition to SLT-homing receptors (CD62L+CCR7+).

Thus, the organization of HSC in BM niches and their retention by CXCL12 raises the fundamental question of whether HSCs and restricted progenitors, including CLP or NKP, reside within distinct, specialized niches or whether they share a common niche. Using CXCL12 knock-in mice and conditional CXCL12 deletion, Ding and Morrison provided evidence that *Cxcl12* was primarily expressed by perivascular stromal cells and at lower levels by endothelial cells, osteoblasts, and some haematopoietic cells (20). Interestingly, deletion of *CXCL12* from endothelial cells depleted HSCs and certain restricted progenitors, but not myeloerythroid or lymphoid-committed progenitors, from perivascular stromal cells, while deletion of *CXCL12* from osteoblasts depleted certain early lymphoid progenitors, but not HSCs (20). Therefore, these findings provided evidence that different stem/progenitor cells occupy distinct cellular niches in BM. Accordingly, while HSCs reside in a perivascular niche, early lymphoid progenitors are localized in an endosteal niche (Figure 1).

This particular organization leaves open the fundamental question of whether the observation of stem/progenitor cells in healthy, steady state conditions actually reflects the whole progenitor potential that is present in the BM and whether this may actually reflect which progenitors/cell types are released from the BM during inflammatory states. Remarkably, support for this question is provided by the observation that chronic inflammation is associated with bone remodeling including endosteal niches, as a result of cytokine-induced modulation of the cells responsible for MMP-9/CXCR4-dependent HSC retention (49, 50). In this regard, proinflammatory cytokines, that include TNFα and IL-1, have been shown to regulate CXCL12 expression, induce lymphocyte mobilization by suppressing CXCL12 retention signals in BM, and to promote the appearance of developing B cells in the spleen (51). Importantly, BM egress could be achieved in the absence of amoeboid migration toward BM exit sites. Accordingly, immature B cell egress from BM has been shown to rely on CXCR4 down-regulation. This passive mode of cell egress from BM also contributes significantly to the export of other hematopoietic cells, including granulocytes, monocytes, and NK cells, and is reminiscent of erythrocyte egress (52).

## Inflammation-Associated Lin^−^CD34^+^DNAM-1^bright^CXCR4^+^ Cell Precursors

Taken together, all the mentioned evidences showing that HSC and CLPs in the BM occupy distinct niches, contribute to generate the concept that BM exit modality in different physiological or pathological conditions differ. Thus, in steady state conditions, mature NK cells and CLP would exit the BM following CXCR4 down-regulation. On the contrary, in the presence of inflammation or inflammatory cytokines, including TNFα, IL-1, and G-CSF, the production of CXCL12 is suppressed and would then allow exit from the BM of lymphocytes lacking CXCR4 down-modulation.

Therefore, the question is whether any “inflammatory” CLP exist, and if so, is the progeny actually superimposable to the one of the so-far characterized CLPs described in BM and other tissues. According to this model and to the interplay between CXCL12 expression and inflammation, CLP and NK cell progenitors released from the BM following inflammatory conditions would be expected to still express CXCR4 (20, 48, 50–52).

Indeed, proof that “inflammatory” or “emergency” progenitors actually exist has been provided by the analysis of patients with chronic inflammatory disorders including patients with HIV-1, chronic HCV infection, TB, as well as COPD or PAPA syndrome (53).

During these inflammatory conditions, Lin^−^CD34^+^DNAM-1^bright^CXCR4^+^ cells can be detected in the PB, where they often represent the majority of CD34+ cells (53). These precursors were found to generate *in vitro* NK cells and T cells but not cells of myelomonocytic lineage under the assayed culture conditions, thus qualifying for a CLP definition. Lin^−^CD34^+^DNAM-1^bright^CXCR4^+^ CLP reside in the BM under steady-state conditions, where they represent 10% of CD34+ cells, while they are not or are poorly (<0.5–1% of PBMC) detectable in PB in healthy, uninflamed conditions (53). The circulating pool size of Lin^−^CD34^+^DNAM-1^bright^CXCR4^+^ cells is significantly increased in patients with inflammatory disorders compared to HD. In cART-treated virologically suppressed HIV-1 patients, for instance, they may represent as much as 30% of Lin^−^ gated PBMC, and over one third of patients had proportions of circulating Lin^−^CD34^+^DNAM-1^bright^CXCR4^+^ cells in excess of 5% of PBMC (53). Notably, the proportion of circulating Lin^−^CD34^+^DNAM-1^bright^CXCR4^+^ correlated directly with fibrinogen concentrations and therefore different output from the BM to the PB likely reflect, among other factors, individual differences in systemic chronic inflammation (53).

In addition to high DNAM-1 and CXCR4 expression (which are absent on conventional CD34+ cells in PB or in UCMC), inflammatory or “emergency” CD34+ CLP express HLA-DR, CD38, CD69, while they do not express CD117, CD94, CD123, CD161. A fraction of these mobilized inflammatory CLP variably express no or very low CD7 (0.2–3%) or CD10 (0.2–10%). Therefore, these CLP represent a heterogeneous population that may well contain small proportions of more committed CD34+CD7+CD10+CD127– single-lineage NK precursors similar to those described recently for classical CD34+ cells (38). These inflammation-dependent CLP, however, predominantly contain less committed precursors upstream the NK cell progenitor fate-decision, which have the potential do develop *in vitro* also to CD3+ T cells and to CD3+CD56+ cells. For example, according to the study by Doulatov et al. (54), Lin^−^CD34^+^DNAM-1^bright^CXCR4^+^ cells would surprisingly also fit in the group of megakaryocyte/erythroid precursors, characterized by the CD38+CD10–CD7–Flt3– phenotype. In addition, their lack of CD127 and CD161 expression could rule out their developmental trajectory toward ILCs. This is confirmed by the lack of ILC growth *in vitro* and ILC-compatible transcription (53). However, there is still room for the possibility to find “inflammatory” ILC precursors, in view of the observation that EILIPs transiently lack CD127 (7, 42), and that so-far uncharacterized Lin^−^CD34^+^DNAM-1^bright^CXCR4^+^ precursors may be observed in infectious/inflammatory conditions.

Importantly, the chemokine receptor expression of Lin^−^CD34^+^DNAM-1^bright^CXCR4^+^ cells is different from the one of conventional circulating Lin^−^CD34^+^DNAM-1^−^CXCR4^−^ CLP. Indeed, while the latter predominantly express CD62L or CCR7 (>90%) and therefore appear to be homing predominantly toward SLT, a relevant proportion (35%) of Lin^−^CD34^+^DNAM-1^bright^CXCR4^+^ cells express CXCR3, CXCR1, or CX3CR1 and therefore appear to be poised to a relevant extent to peripheral inflamed tissues, not only to SLT (53).

A peculiar feature of Lin^−^CD34^+^DNAM-1^bright^CXCR4^+^ cells is represented by the unusual characteristics of their NK and T cell progenies. Under limiting dilution conditions, NK cell progenies grow rapidly and, already 16–20 days after seeding, have acquired a mature NK cell phenotype with expression of Natural Cytotoxicity Receptors (NKp46, NKp30, NKp44), CD244, HLA-DR, NKG2D, DNAM-1, NKG2A/CD94, Killer-like Immunoglobulin Receptors (KIRs) (53). In addition, they express high levels of Perforin, are cytotoxic toward tumor cell lines, and produce abundant IFNγ. The NK cell progeny of Lin^−^CD34^+^DNAM-1^bright^CXCR4^+^ cells, produces IFNγ with an early production pattern (53), which is superimposable to the one observed in mature CD56^dim^ NK cells (34, 53). Since CD56^bright^ NK cells are known to be developmentally upstream of CD56^dim^ NK cells (44, 55) and have a “late” IFNγ production pattern (34), this IFNγ production pattern in the progenies is somehow surprising as one would rather expect a CD56^bright^-like pattern. In addition, this finding is also unusual as classical CD34+DNAM-1-CXCR4- cells generate CD56^bright^-like, poorly functional, maturing NK cells almost exclusively *in vitro*, that have low NCR and very low to absent perforin, NKG2D, DNAM-1 expression and IFNγ production (31, 46). Indeed, NK cells develop from CD34+ precursors following a 4-staged expression of receptors (CD34, CD117, CD94, CD56, CD16), originally described by Freud and coll in CD34+ cells from tonsils and lymph nodes (55), and more recently revised to include six distinct stages (56). In particular, a small fraction of maturing NK cell progenies from “classical” CD34+DNAM-1- precursors may express KIRs only after prolonged culture and IL-21 stimulation (37), while progenies from “inflammatory” Lin^−^CD34^+^DNAM-1^bright^CXCR4^+^ cells in the BM and PB readily express KIRs after 16–20 days of progenitor seeding, and may follow a different staged development (53).

## Concluding Remarks

Thus, “inflammatory” DNAM-1^bright^ CD34^+^CXCR4^+^ CLP (53), distinct from “classical” CD34^+^DNAM-1^−^CXCR4^−^ progenitors (23, 24, 38, 45, 55–57), stably reside in BM at steady state in a presumed osteoblast niche. They do not circulate in PB in easily detectable amounts, but are ready to be rapidly deployed to the peripheral tissues following stimuli that may include inflammation-induced CXCL12 downmodulation (48, 51, 52) (Figure 1B). Recruitment to the periphery includes, for example, G-CSF. Indeed, G-CSF-induced mobilization/harvest protocols for transplantation purposes where Lin^−^CD34^+^DNAM-1^bright^CXCR4^+^ cells are released together with other CD34+ HSC/CLPs (53). Importantly, Lin^−^CD34^+^DNAM-1^bright^CXCR4^+^ cell frequencies in PB prevail over conventional CD34^+^DNAM-1^−^CXCR4^−^ cells during chronic inflammation. This “emergency” deployment may be mechanistically interpreted to support the model of a CD34+ travel trajectory toward inflamed peripheral tissues—in addition to SLT—when an increased turnover of lymphoid cells occurs at sites of infection/inflammation. This would allow the prompt availability of function-ready NK cells with an unconventional mature CD56dim-like functional activity.

Thus, the current views of NK cell development need to take into account recent evidences. Indeed, our views have been upgraded from a strictly hierarchical stepwise organization of fate decisions for HSCs and CLPs toward progressively more restricted maturing potentials (23, 38, 55, 56) to a comprehensive system where HSC and CLP fate decisions are less strict. In this comprehensive system, they depend on more shared transcriptional programs, additional conditions including local tissue signals (e.g., delivered by stromal cells) and system requirements (steady state vs. recolonization) (58–61). Modeling in the presence of increased peripheral turnover with inflammation has so far been assumed to fall into the “steady state,” and has therefore led to the substantial lack of consideration of “inflammatory” CD34+ in studies on NK cell development (24, 54–56). Thus, this explains how these CLPs eluded characterization for a long time. With the demonstration of the regulation of HSC release from distinct niches in the BM (20, 46–48, 50), and the characterization of “inflammatory” CD34+ progenitors in the BM (53), some so-far unanswered questions along the path of NK cell development could be addressed. Indeed, the surprisingly wide spectrum of NK cell phenotypic and functional repertoires (62) still has unanswered aspects, including the origin of tissue-resident NK cells, the origin of such a variety of phenotypic differences, and the exact boundaries for the generation of memory-like NK cells (1, 63–69). Inclusion of Lin^−^CD34^+^DNAM-1^bright^CXCR4^+^ in the modeling of NK cell development in inflamed BM, SLT and peripheral tissues introduces an additional level of complexity to an already full pattern of developmental steps (56, 59), but will help to address some unanswered questions. In view of the ongoing effort at redirecting NK cells for immunotherapeutic purposes (e.g., anti-KIR, anti-NKG2A mAbs, CAR-NK engineering), the existence of inflammatory CD34+DNAM-1^bright^ precursors with extremely functional NCR+NKG2D+ NK cells could represent a useful tool for immunotherapeutic purposes.

## Author Contributions

FB, CP, LM, and AD contributed to writing the manuscript.

### Conflict of Interest Statement

The authors declare that the research was conducted in the absence of any commercial or financial relationships that could be construed as a potential conflict of interest.

## References

[1] Vivier E Raulet DH Moretta A Caligiuri MA Zitvogel L Lanier LL. Innate or adaptive immunity? The example of natural killer cells. Science. (2011) 331:44–9. 10.1126/science.1198687 21212348 PMC3089969

[2] Moretta A. Natural killer cells and dendritic cells: rendezvous in abused tissues. Nat Rev Immunol. (2002) 2:957–65. 10.1038/nri956 12461568

[3] Peppa D Gill US Reynolds G Easom NJ Pallett LJ Schurich A. Up-regulation of a death receptor renders antiviral T cells susceptible to NK cell-mediated deletion. J Exp Med. (2013) 210:99–114. 10.1084/jem.20121172 23254287 PMC3549717

[4] Vargas-Inchaustegui DA Xiao P Tuero I Patterson LJ Robert-Guroff M. NK and CD4+ T cell cooperative immune responses correlate with control of disease in a macaque simian immunodeficiency virus infection model. J Immunol. (2012) 189:1878–85. 10.4049/jimmunol.1201026 22798665 PMC3411935

[5] De Boer RJ Mohri H Ho DD Perelson AS. Turnover rates of B cells, T cells, and NK cells in simian immunodeficiency virus-infected and uninfected rhesus macaques. J Immunol. (2003) 170:2479–87. 10.4049/jimmunol.170.5.2479 12594273

[6] Diefenbach A Colonna M Koyasu S. Development, differentiation, and diversity of innate lymphoid cells. Immunity. (2014) 41:354–65. 10.1016/j.immuni.2014.09.005 25238093 PMC4171710

[7] Cherrier DE Serafini N Di Santo JP. Innate lymphoid cell development: a T cell perspective. Immunity. (2018) 48:1091–103. 10.1016/j.immuni.2018.05.010 29924975

[8] Daussy C Faure F Mayol K Viel S Gasteiger G Charrier E. T-bet and Eomes instruct the development of two distinct natural killer cell lineages in the liver and in the bone marrow. J Exp Med. (2014) 211:563–77. 10.1084/jem.20131560 24516120 PMC3949572

[9] Robinette ML Fuchs A Cortez VS Lee JS Wang Y Durum SK. Transcriptional programs define molecular characteristics of innate lymphoid cell classes and subsets. Nat Immunol. (2015) 16:306–17. 10.1038/ni.3094 25621825 PMC4372143

[10] Artis D Spits H. The biology of innate lymphoid cells. Nature. (2015) 517:293–301. 10.1038/nature14189 25592534

[11] Moretta A Bottino C Vitale M Pende D Cantoni C Mingari MC. Activating receptors and coreceptors involved in human natural killer cell-mediated cytolysis. Annu Rev Immunol. (2001) 19:197–223. 10.1146/annurev.immunol.19.1.197 11244035

[12] Moretta A Biassoni R Bottino C Moretta L. Surface receptors delivering opposite signals regulate the function of human NK cells. Semin Immunol. (2000) 12:129–38. 10.1006/smim.2000.0215 10764621

[13] Moretta A Bottino C Vitale M Pende D Biassoni R Mingari MC. Receptors for HLA class-I molecules in human natural killer cells. Annu Rev Immunol. (1996) 14:619–48. 10.1146/annurev.immunol.14.1.619 8717527

[14] Jacobson LO Simmons EL Marks EK Eldredge JH. Recovery from radiation injury. Science. (1951) 113:510–1. 10.1126/science.113.2940.510 14828383

[15] Till JE Mc CE. A direct measurement of the radiation sensitivity of normal mouse bone marrow cells. Radiat Res. (1961) 14:213–22. 10.2307/3570892 13776896

[16] Berardi AC Wang A Levine JD Lopez P Scadden DT. Functional isolation and characterization of human hematopoietic stem cells. Science. (1995) 267:104–8. 10.1126/science.7528940 7528940

[17] Spangrude GJ Heimfeld S Weissman IL. Purification and characterization of mouse hematopoietic stem cells. Science. (1988) 241:58–62. 10.1126/science.2898810 2898810

[18] Terstappen LW Huang S Safford M Lansdorp PM Loken MR. Sequential generations of hematopoietic colonies derived from single nonlineage-committed CD34+CD38- progenitor cells. Blood. (1991) 77:1218–27. 1705833

[19] Weissman IL Shizuru JA. The origins of the identification and isolation of hematopoietic stem cells, and their capability to induce donor-specific transplantation tolerance and treat autoimmune diseases. Blood. (2008) 112:3543–53. 10.1182/blood-2008-08-078220 18948588 PMC2574516

[20] Ding L Morrison SJ. Haematopoietic stem cells and early lymphoid progenitors occupy distinct bone marrow niches. Nature. (2013) 495:231–5. 10.1038/nature11885 23434755 PMC3600153

[21] Rosen SD. Ligands for L-selectin: homing, inflammation, and beyond. Annu Rev Immunol. (2004) 22: 129–56. 10.1146/annurev.immunol.21.090501.080131 15032576

[22] Vivier E Artis D Colonna M Diefenbach A Di Santo JP Eberl G. Innate lymphoid cells: 10 years on. Cell. (2018) 174:1054–66. 10.1016/j.cell.2018.07.017 30142344

[23] Galy A Travis M Cen D Chen B. Human T, B, natural killer, and dendritic cells arise from a common bone marrow progenitor cell subset. Immunity. (1995) 3:459–73. 10.1016/1074-7613(95)90175-2 7584137

[24] Colucci F Caligiuri MA Di Santo JP. What does it take to make a natural killer? Nat Rev Immunol. (2003) 3:413–25. 10.1038/nri1088 12766763

[25] Passlick B Izbicki JR Waydhas C Nast-Kolb D Schweiberer L Ziegler-Heitbrock HW Posttraumatic splenectomy does not influence human peripheral blood mononuclear cell subsets. J Clin Lab Immunol. (1991) 34:157–61. 1668282

[26] Ramos SB Garcia AB Viana SR Voltarelli JC Falcao RP. Phenotypic and functional evaluation of natural killer cells in thymectomized children. Clin Immunol Immunopathol. (1996) 81:277–81. 10.1006/clin.1996.0189 8938105

[27] Sirianni MC Businco L Seminara R Aiuti F. Severe combined immunodeficiencies, primary T-cell defects and DiGeorge syndrome in humans: characterization by monoclonal antibodies and natural killer cell activity. Clin Immunol Immunopathol. (1983) 28:361–70. 10.1016/0090-1229(83)90103-4 6349883

[28] Kohn LA Hao QL Sasidharan R Parekh C Ge S Zhu Y. Lymphoid priming in human bone marrow begins before expression of CD10 with upregulation of L-selectin. Nat Immunol. (2012) 13:963–71. 10.1038/ni.2405 22941246 PMC3448017

[29] Six EM Bonhomme D Monteiro M Beldjord K Jurkowska M Cordier-Garcia C. A human postnatal lymphoid progenitor capable of circulating and seeding the thymus. J Exp Med. (2007) 204:3085–93. 10.1084/jem.20071003 18070935 PMC2150974

[30] Davi F Faili A Gritti C Blanc C Laurent C Sutton L. Early onset of immunoglobulin heavy chain gene rearrangements in normal human bone marrow CD34+ cells. Blood. (1997) 90:4014–21. 9354670

[31] Verneris MR Miller JS. The phenotypic and functional characteristics of umbilical cord blood and peripheral blood natural killer cells. Br J Haematol. (2009) 147:185–91. 10.1111/j.1365-2141.2009.07768.x 19796267 PMC2770803

[32] Res P Martinez-Caceres E Cristina Jaleco A Staal F Noteboom E Weijer K. CD34+CD38dim cells in the human thymus can differentiate into T, natural killer, and dendritic cells but are distinct from pluripotent stem cells. Blood. (1996) 87:5196–206. 10.1016/S0165-2478(97)85340-0 8652833

[33] McClory S Hughes T Freud AG Briercheck EL Martin C Trimboli AJ. Evidence for a stepwise program of extrathymic T cell development within the human tonsil. J Clin Invest. (2012) 122:1403–15. 10.1172/JCI46125 22378041 PMC3314444

[34] De Maria A Bozzano F Cantoni C Moretta L Revisiting human natural killer cell subset function revealed cytolytic CD56dimCD16+ NK cells as rapid producers of abundant IFN-Œ≥ on activation. Proc Natl Acad Sci USA. (2011) 108:728–32. 10.1073/pnas.1012356108 21187373 PMC3021076

[35] Costa P Sivori S Bozzano F Martini I Moretta A Moretta L. IFN-alpha-mediated increase in cytolytic activity of maturing NK cell upon exposure to HSV-infected myelomonocytes. Eur J Immunol. (2009) 39:147–58. 10.1002/eji.200838532 19089810

[36] Sivori S Falco M Marcenaro E Parolini S Biassoni R Bottino C. Early expression of triggering receptors and regulatory role of 2B4 in human natural killer cell precursors undergoing *in vitro* differentiation. Proc Natl Acad Sci USA. (2002) 99:4526–31. 10.1073/pnas.072065999 11917118 PMC123681

[37] Sivori S Cantoni C Parolini S Marcenaro E Conte R Moretta L. IL-21 induces both rapid maturation of human CD34+ cell precursors towards NK cells and acquisition of surface killer Ig-like receptors. Eur J Immunol. (2003) 33:3439–47. 10.1002/eji.200324533 14635054

[38] Renoux VM Zriwil A Peitzsch C Michaelsson J Friberg D Soneji S. Identification of a human natural killer cell lineage-restricted progenitor in fetal and adult tissues. Immunity. (2015) 43:394–407. 10.1016/j.immuni.2015.07.011 26287684

[39] Montaldo E Teixeira-Alves LG Glatzer T Durek P Stervbo U Hamann W. Human RORgammat(+)CD34(+) cells are lineage-specified progenitors of group 3 RORgammat(+) innate lymphoid cells. Immunity. (2014) 41:988–1000. 10.1016/j.immuni.2014.11.010 25500367

[40] Tang Q Ahn YO Southern P Blazar BR Miller JS Verneris MR. Development of IL-22-producing NK lineage cells from umbilical cord blood hematopoietic stem cells in the absence of secondary lymphoid tissue. Blood. (2011) 117:4052–5. 10.1182/blood-2010-09-303081 21310921 PMC3087531

[41] Yang Q Li F Harly C Xing S Ye L Xia X. TCF-1 upregulation identifies early innate lymphoid progenitors in the bone marrow. Nat Immunol. (2015) 16:1044–50. 10.1038/ni.3248 26280998 PMC4575643

[42] Klose Christoph SN Flach M Möhle L Rogell L Hoyler T Ebert K. Differentiation of type 1 ILCs from a common progenitor to all helper-like innate lymphoid cell lineages. Cell. (2014) 157:340–56. 10.1016/j.cell.2014.03.030 24725403

[43] Fehniger TA Cooper MA Nuovo GJ Cella M Facchetti F Colonna M. CD56bright natural killer cells are present in human lymph nodes and are activated by T cell-derived IL-2: a potential new link between adaptive and innate immunity. Blood. (2003) 101:3052–7. 10.1182/blood-2002-09-2876 12480696

[44] Ferlazzo G Thomas D Lin SL Goodman K Morandi B Muller WA. The abundant NK cells in human secondary lymphoid tissues require activation to express killer cell Ig-like receptors and become cytolytic. J Immunol. (2004) 172:1455–62. 10.4049/jimmunol.172.3.1455 14734722

[45] Freud AG Becknell B Roychowdhury S Mao HC Ferketich AK Nuovo GJ. A human CD34(+) subset resides in lymph nodes and differentiates into CD56bright natural killer cells. Immunity. (2005) 22:295–304. 10.1016/j.immuni.2005.01.013 15780987

[46] Ding L Saunders TL Enikolopov G Morrison SJ. Endothelial and perivascular cells maintain haematopoietic stem cells. Nature. (2012) 481:457–62. 10.1038/nature10783 22281595 PMC3270376

[47] Kunisaki Y Bruns I Scheiermann C Ahmed J Pinho S Zhang D. Arteriolar niches maintain haematopoietic stem cell quiescence. Nature. (2013) 502:637–43. 10.1038/nature12612 24107994 PMC3821873

[48] Greenbaum A Hsu Y-MS Day RB Schuettpelz LG Christopher MJ Borgerding JN. CXCL12 in early mesenchymal progenitors is required for haematopoietic stem-cell maintenance. Nature. (2013) 495:227–30. 10.1038/nature11926 23434756 PMC3600148

[49] Takayanagi H. Osteoimmunology: shared mechanisms and crosstalk between the immune and bone systems. Nat Rev Immunol. (2007) 7:292–304. 10.1038/nri2062 17380158

[50] Kollet O Dar A Shivtiel S Kalinkovich A Lapid K Sztainberg Y. Osteoclasts degrade endosteal components and promote mobilization of hematopoietic progenitor cells. Nat Med. (2006) 12:657–64. 10.1038/nm1417 16715089

[51] Ueda Y Yang K Foster SJ Kondo M Kelsoe G. Inflammation Controls B Lymphopoiesis by Regulating Chemokine CXCL12 Expression. J Exp Med. (2004) 199:47–58. 10.1084/jem.20031104 14707114 PMC1887733

[52] Beck TC Gomes AC Cyster JG Pereira JP. CXCR4 and a cell-extrinsic mechanism control immature B lymphocyte egress from bone marrow. J Exp Med. (2014) 211:2567–81. 10.1084/jem.20140457 25403444 PMC4267240

[53] Bozzano F Marras F Ascierto ML Cantoni C Cenderello G Dentone C. ‘Emergency exit’ of bone-marrow-resident CD34(+)DNAM-1(bright)CXCR4(+)-committed lymphoid precursors during chronic infection and inflammation. Nat Commun. (2015) 6:8109. 10.1038/ncomms9109 26436997 PMC4600731

[54] Doulatov S Notta F Eppert K Nguyen LT Ohashi PS Dick JE. Revised map of the human progenitor hierarchy shows the origin of macrophages and dendritic cells in early lymphoid development. Nat Immunol. (2010) 11:585–93. 10.1038/ni.1889 20543838

[55] Freud AG Yokohama A Becknell B Lee MT Mao HC Ferketich AK. Evidence for discrete stages of human natural killer cell differentiation *in vivo*. J Exp Med. (2006) 203:1033–43. 10.1084/jem.20052507 16606675 PMC2118285

[56] Scoville SD Freud AG Caligiuri MA. Modeling human natural killer cell development in the era of innate lymphoid cells. Front Immunol. (2017) 8:360. 10.3389/fimmu.2017.00360 28396671 PMC5366880

[57] Eissens DN Spanholtz J van der Meer A van Cranenbroek B Dolstra H Kwekkeboom J. Defining early human NK cell developmental stages in primary and secondary lymphoid tissues. PLoS ONE. (2012) 7:e30930. 10.1371/journal.pone.0030930 22319595 PMC3272048

[58] Notta F Zandi S Takayama N Dobson S Gan OI Wilson G. Distinct routes of lineage development reshape the human blood hierarchy across ontogeny. Science. (2016) 351:aab2116. 10.1126/science.aab2116 26541609 PMC4816201

[59] Laurenti E Gottgens B. From haematopoietic stem cells to complex differentiation landscapes. Nature. (2018) 553:418–26. 10.1038/nature25022 29364285 PMC6555401

[60] Laurenti E Doulatov S Zandi S Plumb I Chen J April C. The transcriptional architecture of early human hematopoiesis identifies multilevel control of lymphoid commitment. Nat Immunol. (2013) 14:756–63. 10.1038/ni.2615 23708252 PMC4961471

[61] Carrelha J Meng Y Kettyle LM Luis TC Norfo R Alcolea V. Hierarchically related lineage-restricted fates of multipotent haematopoietic stem cells. Nature. (2018) 554:106–111. 10.1038/nature25455 29298288

[62] Horowitz A Strauss-Albee DM Leipold M Kubo J Nemat-Gorgani N Dogan OC. Genetic and environmental determinants of human NK cell diversity revealed by mass cytometry. Sci Transl Med. (2013) 5:208ra145. 10.1126/scitranslmed.3006702 24154599 PMC3918221

[63] Freud AG Mundy-Bosse BL Yu J Caligiuri MA. The broad spectrum of human natural killer cell diversity. Immunity. (2017) 47:820–33. 10.1016/j.immuni.2017.10.008 29166586 PMC5728700

[64] Aw Yeang HX Piersma SJ Lin Y Yang L Malkova ON Miner C. Cutting edge: human CD49e-NK cells are tissue resident in the liver. J Immunol. (2017) 198:1417–22. 10.4049/jimmunol.1601818 28093522 PMC5296254

[65] Cuff AO Robertson FP Stegmann KA Pallett LJ Maini MK Davidson BR Eomes-NK cells in human liver are long-lived and do not recirculate but can be replenished from the circulation. J Immunol. (2016) 197:4283–91. 10.4049/jimmunol.1601424 27798170 PMC5114885

[66] Stegmann KA Robertson F Hansi N Gill U Pallant C Christophides T. CXCR6 marks a novel subset of T-bet(lo)Eomes(hi) natural killer cells residing in human liver. Sci Rep. (2016) 6:26157. 10.1038/srep26157 27210614 PMC4876507

[67] Sun JC Beilke JN Lanier LL. Adaptive immune features of natural killer cells. Nature. (2009) 457:557–61. 10.1038/nature07665 19136945 PMC2674434

[68] Guma M Budt M Saez A Brckalo T Hengel H Angulo A. Expansion of CD94/NKG2C+ NK cells in response to human cytomegalovirus-infected fibroblasts. Blood. (2006) 107:3624–31. 10.1182/blood-2005-09-3682 16384928

[69] Vacca P Vitale C Montaldo E Conte R Cantoni C Fulcheri E. CD34+ hematopoietic precursors are present in human decidua and differentiate into natural killer cells upon interaction with stromal cells. Proc Natl Acad Sci USA. (2011) 108:2402–7. 10.1073/pnas.1016257108 21248224 PMC3038730

